# The effect of sulforaphane on markers of inflammation and metabolism in virally suppressed HIV patients

**DOI:** 10.3389/fnut.2024.1357906

**Published:** 2024-10-30

**Authors:** Jose Giron, Lauren Smiarowski, Johannah Katz

**Affiliations:** ^1^Sunshine Specialty Health Care, Orlando, FL, United States; ^2^Department of Medicine, Florida State University, College of Medicine, Tallahassee, FL, United States; ^3^Department of Medicine, University of Central Florida College of Medicine, Orlando, FL, United States; ^4^OrlandoHealth- Arnold Palmer Hospital for Children, Orlando, FL, United States; ^5^Florida Center for Hormones and Wellness, Orlando, FL, United States

**Keywords:** human immunodeficiency virus, sulforaphane (cruciferous antioxidants), antioxidant — phytochemical studies, metabolic syndome, acquired immunodeficiency syndrome, highly active antiretroviral therapy

## Abstract

There are currently 1.2 million people living with HIV (Human Immunodeficiency Virus) in the United States. Virally suppressed HIV patients commonly experience chronic inflammation which increases the risk for other chronic conditions. This inflammation can be quantified with a variety of biomarkers. Some current antiretroviral compounds bring about metabolic abnormalities and promote weight gain often associated with increases in visceral adipose tissue (VAT) and an increase in the risk of diabetes mellitus and cardiovascular disease. Sulforaphane, an isothiocyanate found in cruciferous vegetables, has shown efficacy in animal models by reducing lipid levels, lowering inflammatory markers, and decreasing fat mass. A double-blind randomized controlled pilot study with 14 virally suppressed HIV patients was conducted to evaluate the effects of 40 mg (225 μmol) of sulforaphane, once daily, over 12 weeks, followed by a 4-week washout period. There was a significant decrease in C-reactive protein compared to the control group (*p* = 0.019). Sulforaphane has been studied in a multitude of conditions and diseases, but this is the first study in a human population of patients living with HIV.

## Introduction

There are currently 1.2 million people living with HIV (Human Immunodeficiency Virus) in the United States (1). Therapeutic advances in HIV care have dramatically altered the natural history of HIV disease allowing patients to live normal lives. Although life expectancy has significantly increased, it remains slightly less than the general population- one theory for this is related to chronic inflammation. Patients living with virally suppressed HIV exist in a state of chronic inflammation, quantifiable by inflammatory markers such as Interleukin-6 (IL-6) and C-reactive protein (CRP) (2). Living in a state of chronic inflammation increases the risk for other chronic conditions in these patients. A commonly experienced side effect of highly active antiretroviral therapies (HAART) is metabolic abnormalities such as increases in lipids, triglycerides, and weight (3). These changes are associated with increases in visceral adipose tissue (VAT), which in turn increase the risk of diabetes and cardiovascular disease (CVD) (3–5).

Sulforaphane, a phytochemical, or phytonutrient, and the most potent natural activator of the transcription factor nuclear factor erythroid 2 (Nrf2)- an isothiocyanate produced from the precursor glucoraphanin (GR). It is commonly found in cruciferous vegetables, primarily broccoli, and has been shown to reduce lipid levels, inflammatory markers, and weight in animal models (6). Human studies of sulforaphane have shown improvement in inflammatory markers, and decreasing adipose mass, with an excellent safety profile (7–13).

Sulforaphane has been studied in a variety of clinical settings, both in healthy subjects and in studies of people living with chronic diseases such as hypertension, schizophrenia, type 2 diabetes, fatty liver, sickle cell disease, and asthma (14). Sulforaphane is a bioactive phytochemical, a constituent of plants that in human beings is an activator of cellular defense mechanisms (15). A tremendous body of evidence has now been built around the multiple molecular mediators of these effects, and interventions with glucoraphanin-rich or sulforaphane-rich broccoli preparations in humans lead to diverse beneficial effects (16). Sulforaphane has been shown to reduce inflammatory biomarkers and fat mass in humans and has been shown to ameliorate obesity in animal studies as well (6). Shehatou and Suddek (17), they found sulforaphane supplementation attenuated the development of artherosclerosis and prevented CRP elevation in high cholesterol diets in animal studies. An often-monitored inflammatory marker in these studies is high sensitivity C-reactive protein (HsCRP). CRP is secreted by the liver in response to inflammation in the body in response to inflammatory cytokines. CRP levels rise steeply with acute insult in response to trauma, inflammation, or infection (18). Sulforaphane has not yet been studied in humans living with HIV.

Previous studies investigating Antiretroviral Therapies (ART) has well characterized adverse effects of these disease altering medications. Frequently used medications in the treatment of HIV are Biktarvy, Genvoya, Descovy, Odefsey, and Symtuza all of which are combination medications of two or more antiretrovirals. A meta-analysis examining side effect profiles of ART in 8 randomized clinical trials showed significant weight gain after initiation of Antiretroviral Therapy (ART), in patients living with HIV, particularly with medications including tenofovir alafenamide (TAF) (5). A switch study by Mallon et al. (19) consisting of 6,908 people showed an association with early pronounced weight gain when switching from tenofovir disoproxil fumarate (TDF) to TAF, regardless of which medication was used, which then slowed down approximately 9 months after switching to TAF (19). Along with weight gain, Visceral Adipose Tissue (VAT) accumulation is a major correlate of diabetogenic, atherogenic, prothrombotic, and proinflammatory metabolic abnormalities referred to as metabolic syndrome (20, 23). According to the World Health Organization, 70% of all deaths globally can be attributed to chronic inflammatory diseases. Chronic inflammation has a significant impact on quality of life and increased risk of diseases, as cited in a review by Mazarakis et al. (21), who also described the positive effects of sulforaphane on inflammation.

The aim of the present study was to assess the effects of sulforaphane on inflammatory and lipid biomarkers in a virally suppressed HIV population.

## Methods

### Sampling

This study was approved by the Institutional Review Board of the Florida State University (STUDY00002532). This study was registered with the Food and Drug Administration (FDA) as a clinical trial (NCT05224492) with sulforaphane registered as a new drug (IND: 157470). All 14 participants were virally suppressed HIV patients from a private infectious disease practice directed by one of the authors (JG) in the greater Orlando area. Eligability criteria for this study included: a patient of the JG, living with virally suppressed HIV, demonstrating previous laboratory evidence of metabolic disease. All participants were provided written informed consent to participate in the study. The body mass index (BMI) of all participants had increased by 10% or more since initiation of HIV treatment, and at initiation of study the mean BMI was ≥30. Additional demographic information is available in Table 1.

**Table 1:** Enrolled patient demographics – total 9 patients.

Baseline characteristic	Sulforaphane		Placebo	
	*n = 4*	%	*n = 5*	%
Age				
31–50 years	1	25	3	60
51–70 years	3	75	2	40
Gender				
Female	0	0	1	20
Male	4	100	4	80
Self-identified race/ethnicity				
Hispanic	2	50	3	60
African American	1	25	1	20
Caucasian	1	25	1	20
Sexual behaviors				
MSM	4	100	4	80
No MSM	0	0	1	20

Participants were randomly assigned to either a control group who received a placebo sachet filled with mustard seed powder and maltodextrin or an experimental group who received a dietary supplement consisting of powdered, concentrated glucoraphanin-rich broccoli seed extract and mustard seed powder containing active myrosinase, which react together to produce sulforaphane (hereinafter referred to as “sulforaphane”). Both participants and investigators were blinded to the contents of the product used. Participants received treatment (supplement) or control for 12 weeks. Participants were assigned a participant number, and numbers were randomized via an online random number generator.

After study initiation, investigators measured clinical parameters and recorded any adverse events at intervals of 6, 12, and at 16 weeks after a 4-week washout period.

### Supplementation

The supplements were provided in individual powder sachets for the patient to open and mix into their choice of food or beverage once daily. Both the sulforaphane and the placebo powders were provided by Brassica Protection Products, LLC (Baltimore, MD, United States). Each subject in the experimental group received 1,516 mg of the GR-rich extract (TrueBroc®) plus 601 mg of myrosinase-containing mustard seed powder, calculated to yield approximately 40 mg (225 μmol) of sulforaphane, daily. The placebo group did not receive any sulforaphane as their powder lacked glucoraphanin, the precursor for sulforaphane (14). The TrueBroc compound mixed with mustard seed powder was a beige-yellow color. The mustard seed powder only sachet was the same beige-yellow in color with no discernable difference between the experimental supplement and placebo. Patients in both groups were advised to continue their regular diet throughout the study period. The intervention period was 12 weeks followed by a 4-week washout period.

### Parameters measured

Parameters tested at initiation of the supplement (listed below) provided a baseline for all levels measured throughout the study. HIV viral load was measured to ensure there was no unexpected drug interaction with patients’ existing antiretroviral regimen. Additional safety parameters were measured, including AST, ALT, CBC, CMP, creatinine, CD4 and CD8 to monitor for adverse events related to supplementation. A table of all laboratory tests collected is found in Table 2. Blood specimens for all parameters except for IL-6 were processed through Quest Laboratories or Labcorp. IL-6 samples were processed by an academic lab. During transportation and storage, IL-6 samples became hemolyzed and developed fibrin clots. These samples were deemed unreliable due to sample quality and are thus not included in our results. Adherence to the study was self-reported by participants weekly during a weekly phone check in with one of the investigators. During these calls, participants were asked about if they missed any doses and any side effects they may have experienced. Participants were additionally asked about any changes to their health during the study time period including acute infections and changes to medications.

**Table 2:** List of parameters measured for patients enrolled in the study.

1. White cell count 2. Hemoglobin 3. Platelet count 4. Creatinine 5. AST 6. ALT 7. CD4 8. CD8 9. HIV viral load 10. CRP 11. IL-6 12. Triglycerides 13. Total Cholesterol 14. Low density lipoprotein (LDL) Cholesterol 15. Pregnancy test (for women of childbearing age)

### Statistical analysis

Data were analyzed using T-test assuming unequal variances using percentage change. Data collection and analysis was conducted using Excel (Microsoft V. 16.75) and the alpha was set at 0.05% for determination of significance. Difference in difference value was also calculated for CRP data using the mean CRP at week 0 and mean CRP at week 12. Data are presented in the subsequent tables.

## Results

A total of 9 participants completed this study. A breakdown of their demographic data can be found in Table 1.

### C-reactive protein

Both groups had a reduction in mean C-Reactive Protein (CRP) at the end of 12 weeks, however the sulforaphane group had a significantly greater reduction. Subjects in the sulforaphane group experienced a mean reduction in CRP of 40% at the end of 12 weeks (Table 3), whereas subjects in the placebo group experienced a 12% reduction in CRP at the end of the study intervention. The difference in difference estimate between the two groups at the end of the 12 weeks was −0.61 (*p* = 0.32). Between cessation of sulforaphane treatment at 12 weeks, and the 16-week follow-up visits, the intervention group’s CRP levels increased by 13%. While a 13% increase was seen following cessation of sulforaphane, a non-significant decrease of approximately 21% was observed as compared to baseline. At the 16-week mark, a decrease of 6% was observed in the control group.

**Table 3:** Average change in biomarkers with 12-week supplementation of sulforaphane vs. placebo among adult patients with HIV (*N* = 9).

Parameters (change from baseline)	Treatment (*n* = 4)	Placebo (*n* = 5)	*p*-value (*α* = 0.05)*
CRP			
6 weeks	8.6%	3.6%	
12 weeks	−40%	−11.6%	0.02
16 weeks	−27%	−13%	
Total Cholesterol			
6 weeks	2.0%	0%	
12 weeks	−12%	0%	0.17
16 weeks	−10%	0%	
LDL-C			
6 weeks	−12.8%	−3%	
12 weeks	−18%	−3%	0.24
16 weeks	−24.4%	14%	
Triglycerides			
6 weeks	−15%	−1.9%	
12 weeks	−11%	6.3%	0.21
16 weeks	25.6%	−9.7%	

*p-values calculated from *t*-test.

### Total cholesterol

At 12 weeks, a reduction in total cholesterol (Total-C; Table 3) of 12% was observed in the sulforaphane group while the placebo group experienced no change.

### Low density lipoprotein-C

After 6 weeks of intervention, the low-density lipoprotein-C (LDL-C) levels of the sulforaphane group dropped 12% from baseline levels, and the placebo group dropped only 3% (Table 3). At the end of the intervention period (12 weeks), the intervention group’s LDL-C had dropped by 24%, a differential that was maintained throughout the washout period. Control group LDL-C dropped only 3% by the end of the intervention, though after the washout period, they had a 14% reduction in LDL-C compared to baseline.

### Triglycerides

The average change in triglycerides from baseline to conclusion of the intervention at 12 weeks for the treatment group was −11% (Table 3). The average percent change per patient from baseline to 12 weeks in the placebo group was 6.3% increase.

### Body mass and abdominal circumference

Neither the control nor the experimental group experienced a significant change in weight. Additionally, there was no significant change in abdominal circumference between study groups as demonstrated in Table 4. One waist circumference value is missing from the data.

**Table 4:** Average change from baseline in physical measurements with 12-week supplementation of sulforaphane vs. placebo among adult patients with HIV (*N* = 9).

Parameters (change from baseline)	Treatment (*n* = 3)	Placebo (*n* = 5)	*p*- value (*α* = 0.05)
Weight			
12 weeks	−2%	−1%	0.29
Abdominal circumference			
12 weeks	1%	−3.7%	0.84

### Adverse effects

Patients were contacted weekly via phone to follow up, ensure adherence with the study protocol, and to record any adverse events reported by patients. There were no serious adverse events experienced by patients. Overall, the sulforaphane supplement was well tolerated among study participants. The most frequent reported event was mild nausea (*n* = 2). Two patients reported an episode of emesis. One of the participants chose to drop out of the study due to unrelated health issues. Consistent with the literature, among the patients who did experience nausea, it was brief and subsided over time with continued use. No adverse drug interactions were found with the HIV medications. All side effects and patient experiences were reported to the FDA. Side effects can be found in Appendix A.

## Discussion

This small pilot study shows promise for the efficacy of sulforaphane, a natural and safe compound, in reducing CRP, a key biomarker of chronic inflammation, in patients living with virally suppressed HIV. As chronic inflammation has been well-described as a major contributor to the development of many chronic diseases, we believe interventions to reduce inflammatory stimuli result in beneficial effects in virally suppressed HIV patients (21). No serious adverse events were observed during the course of study. Adverse effects were recorded in Appendix A.

In animal models and in human studies, sulforaphane has shown efficacy in reducing decreasing inflammatory markers through reported decreased activation of NF-kB regulated inflammatory pathways (6–8, 22). Human studies using sulforaphane have demonstrated significant reductions in inflammation in various disease states (eg. Obesity) and in healthy volunteers (11, 12). To the researchers’ knowledge, this is the first study to assess the effect of sulforaphane in humans living with virally suppressed HIV. Patients receiving sulforaphane supplementation in this study demonstrated a significant decrease of 40% in the inflammatory marker C-reactive protein (CRP) from onset of the study (week 0) to conclusion of the supplementation (week 12) (*p* = 0.02). A difference in difference calculation for CRP, found compared to the control group, the sulforaphane group had a decrease of 0.611 mg/dL. During the study period, patients did not report any acute infections that may have otherwise influenced a change in CRP. Patients were monitored throughout the duration of the study for changes in their health including acute infection and changes in medications through weekly phone calls with one of the investigators. With CRP being the only statistically significant change appreciated during the study period as well as the only biomarker measured, unreported changes to a patient’s health could significantly impact the findings of this study. Our findings are consistent with previous research in humans on the use of sulforaphane’s effects on human biomarkers of systemic inflammation.

Virally suppressed HIV patients are commonly in a chronic inflammatory state, which can be quantified by inflammatory markers (2), increasing risks for other chronic conditions. Some of the new antiretroviral compounds bring about metabolic abnormalities (changes in lipids and triglycerides) and promote weight gain often associated with an increase in visceral adipose tissue (3, 5, 24), increasing the risk of diabetes and cardiovascular disease (4). Although a greater decrease in LDL-C was observed in the group supplemented with sulforaphane, this result was non-significant (*p* = 0.24). This decrease in lipids observed in the intervention group is consistent with an effect previously observed in other human studies (7, 8). A decrease in triglycerides was also appreciated during our study, suggesting sulforaphane may induce a moderate, though non-significant (*p* = 0.21) reduction of triglycerides. Further studies with greater study power are warranted to further explore the relationship between sulforaphane, LDL-C, and triglycerides in patients living with HIV.

While our results suggest an improvement in biomarkers in patients living with HIV, our study found little effect on the patient’s body habitus- both weight and abdominal circumference. The sulforaphane group experienced a − 2% change in weight from baseline to 12 weeks, while the placebo group experienced a − 1% change. Additionally, there was no significant change in abdominal circumference between study groups (*p* = 0.84). Studies using larger sample sizes and/or longer interventions are warranted to increase the power of the study, allowing significant trends to be more easily elucidated.

We believe that further, larger studies evaluating the effects of sulforaphane in the HIV population are warranted for sulforaphane as a complement to standard of care for antiretroviral therapy, given the observed significant reduction in CRP, a key biomarker of chronic inflammation, and positive trends for lipid parameters in this small pilot study. We would encourage future studies to include other well-established inflammatory markers to further demonstrate the efficacy of sulforaphane as an effective means of improving inflammation. Longer interventions would also indicate safety profile, effect, and compliance of long-term use.

A limitation to this study is the small sample size as it is a pilot study. As the two arms of this study each contain fewer than 5 participants, the weight of each participant greatly affected the statistical analysis. For this reason, results should be interpreted with caution. Furthermore, with further studies, additional demographic information including education level should be collected by investigators as a proxy for socioeconomic status. A validated measure to collect demographic data should be used in future studies to better characterize patient race and ethnic information. Future studies should incorporate participants keeping a food log to evaluate for any confounding variables in the participants diet such as consuming large quantities of GR rich foods. This study lacked access to a DEXA scan, Tanita technology, or CT slice for most accurate description of body fat mass to better characterize patient body composition. Alternatively, waist circumference was measured as an indicator of visceral adipose tissue, but due to the relatively short timeframe of the intervention it may have been unrealistic to expect changes in this parameter. Furthermore, the presence of mustard seed powder in the placebo (used for taste/smell masking, as well as to ensure greatest fidelity to the active intervention), will invariably contain the isothiocyanates(s) (ITC) specific to mustard seed, almost exclusively allyl-ITC, which is much less active in most pathways investigated, but by-and-large does have similar targets to sulforaphane. Of great importance, the only available biomarker in this study was CRP as the blood samples for IL-8 were hemolyzed during transit. Further studies should incorporate additional biomarkers such as IL-8 to better understand other biomarkers affected in the inflammatory pathway.

## Conclusion

In summary, this pilot study underscores the evidence for the therapeutic effects of sulforaphane in attenuating inflammation and indicates a trend for improving lipid parameters in HIV patients. The intricate interplay between chronic inflammation and metabolic disease in this population emphasizes the need for novel and holistic approaches to management of HIV drug-related side effects. Larger randomized controlled trials are warranted to validate sulforaphane as an adjunctive therapy in the comprehensive care of individuals with HIV. To our knowledge, this is the first study to investigate the effects of sulforaphane on chronic inflammation in HIV patients. Despite the small sample size of this pilot study, *n* = 9, we observed a 40% reduction in CRP over the 12 weeks of the study period, indicating sulforaphane has the potential to be a useful tool in modulating the negative effects of inflammation in virally suppressed HIV patients and potentially in other populations experiencing chronic inflammation.

## Data Availability

The raw data supporting the conclusions of this article will be made available by the authors, without undue reservation.

## Appendix A

**Table A1:** Summary of adverse events reported by patients.

Symptom experienced	Number of patients reporting symptom	Week of study reported	Study arm	Comments
Nausea	2	Weeks 1, 2, and 7	Sulforaphane	
Vomiting	1	Week 1	Sulforaphane	Patient withdrew from the study
Kidney stones	1	Week 3	Control	Patient withdrew from the study
